# Multiple signaling factors and drugs alleviate neuronal death induced by expression of human and zebrafish tau proteins in vivo

**DOI:** 10.1186/s12929-016-0237-4

**Published:** 2016-02-06

**Authors:** Bo-Kai Wu, Rey-Yue Yuan, Huang-Wei Lien, Chin-Chun Hung, Pung-Pung Hwang, Rita Pei-Yeh Chen, Chun-Che Chang, Yung-Feng Liao, Chang-Jen Huang

**Affiliations:** Institute of Biotechnology, National Taiwan University, Taipei, 106 Taiwan; Institute of Biological Chemistry, Academia Sinica, 128 Academia Rd., Sec. 2, Taipei, 115 Taiwan; Department of Neurology, School of Medicine, College of Medicine, Taipei Medical University, Taipei, 110 Taiwan; Institute of Cellular and Organismic Biology, Academia Sinica, Taipei, 115 Taiwan; Department of Entomology, National Taiwan University, Taipei, 106 Taiwan

**Keywords:** Tauopathy, Bcl2-L1, Nrf2, Neurotoxicity, GDNF, Zebrafish

## Abstract

**Background:**

The axonal tau protein is a tubulin-binding protein, which plays important roles in the formation and stability of the microtubule. Mutations in the tau gene are associated with familial forms of frontotemporal dementia with Parkinsonism linked to chromosome-17 (FTDP-17). Paired helical filaments of tau and extracellular plaques containing beta-amyloid are found in the brain of Alzheimer’s disease (AD) patients.

**Results:**

Transgenic models, including those of zebrafish, have been employed to elucidate the mechanisms by which tau protein causes neurodegeneration. In this study, a transient expression system was established to express GFP fusion proteins of zebrafish and human tau under the control of a neuron-specific HuC promoter. Approximately ten neuronal cells expressing tau-GFP in zebrafish embryos were directly imaged and traced by time-lapse recording, in order to evaluate the neurotoxicity induced by tau-GFP proteins. Expression of tau-GFP was observed to cause high levels of neuronal death. However, multiple signaling factors, such as Bcl2-L1, Nrf2, and GDNF, were found to effectively protect neuronal cells expressing tau-GFP from death. Treatment with chemical compounds that exert anti-oxidative or neurotrophic effects also resulted in a similar protective effect and maintained human tau-GFP protein in a phosphorylated state, as detected by antibodies pT212 and AT8.

**Conclusions:**

The novel finding of this study is that we established an expression system expressing tau-GFP in zebrafish embryos were directly imaged and traced by time-lapse recording to evaluate the neurotoxicity induced by tau-GFP proteins. This system may serve as an efficient in vivo imaging platform for the discovery of novel drugs against tauopathy.

## Background

The axonal tau protein is a tubulin-binding protein that plays important roles in the formation and stability of the microtubule (MT) [1, 2]. The status of tau phosphorylation is directly related to its biological activity [3]. Hyperphosphorylated tau has lower affinity for MTs, which results in destabilization of MTs [4]. Tauopathies, a class of neurodegenerative disorders, are known to link to tau aggregates [5]. Mutations in the tau gene are associated with familial forms of frontotemporal dementia with Parkinsonism linked to chromosome-17 (FTDP-17) [6, 7]. Frontotemporal dementia (FTD) is an adult neurodegenerative disorder that exhibits symptoms commonly seen in tauopathy-associated dementia. Alzheimer’s disease (AD) belongs to one tauopathy family, and is characterized by the presence of intracellular neurofibrillary tangles (NFTs) composed of paired helical filaments of tau, and extracellular plaques containing beta-amyloid [8, 9].

Truncated forms of tau protein [10] are also found in NFTs in AD brain, suggesting that truncated tau may predispose toward the formation of NFTs [11–14]. Truncation of tau protein has been identified in human sporadic AD [12], and proteolytic cleavage of tau has been proposed to be an early event in the aggregation of tau protein and formation of neurofibrillary lesions in AD. Caspases and other proteases were found to cleave human tau protein at discrete locations in vivo; these sites include Asp^421^ [15–17] and Glu^391^ [10]. Purified recombinant tau proteins truncated after Glu^391^ or Asp^421^ (i.e., hTau-△392 or hTau-△422) aggregate easily in vitro, resulting in more rapid fibrillization than that of intact proteins [17, 18]. In addition, the caspase-3-cleaved tau fragment was demonstrated to propagate the formation of AD-like NFTs in a transgenic rat model [14], corroborating the causative role of truncated tau in AD neurodegeneration in vivo.

Human tau is encoded by the *MAPT* (microtubule-associated protein tau) gene with 16 exons. In the adult human brain, six isoforms of the tau gene, which are products of alternative splicing of exons 2, 3, and 10, have been identified. Three isoforms have three tubulin-binding domains (3R), and the other three isoforms (4R) have an additional tubulin-binding domain encoded by exon 10 [19, 20]. Inclusion of exon 2, or exons 2 and 3, gives rise to an additional 29 or 58 amino acids at the N-terminal region, respectively [21]. In zebrafish, two *MAPT* paralogous genes, *mapta* and *maptb*, have been identified [22] and are regarded to be derived from an ancestral allele of teleost *MAPT* by duplication. Spliced transcripts from both genes indicated that isoforms of *mapta* encode four, five, or six tubulin-binding repeats (4R-6R), while those of *maptb* are mainly the 3R isoforms. Expression of both genes is predominantly observed in the developing central nervous system (CNS), suggesting that they possess essential roles in the embryonic development of the CNS.

Transgenic models such as mice (*Mus musculus*) or flies (*Drosophila melanogaster*) with human *tau* genes allow elucidation of how tau protein causes neurodegeneration in tauopathies. In *Drosophila*, induction of either the endogenous tau or the ectopic human tau can result in neurodegeneration tau [23, 24]. In addition, overexpressing human tau in *Drosophila* leads to a profound disruption of neuronal function prior to the emergence of neurodegeneration [25, 26]. These data thus suggest that the neurotoxic effects of tau are evolutionarily conserved.

Zebrafish (*Danio rerio*) has been demonstrated as an excellent genetic model for studying vertebrate development and diseases [27]. Taking such advantages, a zebrafish model of tauopathy was made for expressing human tau-GFP fusion protein under the control of a neuronal enhancer derived from the gata2 promoter [28]. However, the neurotoxicity of the tau-GFP fusion protein prevented the establishment of stable transgenic lines. Another transgenic zebrafish model of tauopathy was generated by expressing human tau using the zebrafish enolase2 promoter [29]. Although this line could specifically express human tau protein in the nervous system, no significant tauopathy was observed at larval stages of development. This may be due to week expression of the enolase gene in the first 60 h post fertilization. Recently, a Gal4-UAS-based zebrafish model of tauopathy was created for expressing human Tau-P301L [30]. A neuronal HuC promoter [31] was used to drive the expression of this mutant 4R-tau protein. This model recapitulates some of the most important pathological features of tauopathies and has been adopted for drug screening based on whole-mount antibody staining using different anti-tau conformation antibodies. However, this procedure is time-consuming, and direct image tracing is difficult to perform.

In order to improve the assay of human tau in zebrafish, we generated a model where tau-GFP expression and neurotoxicity could be live monitored. We found that neuronal cells expressing tau-GFP in these models can be readily imaged and traced to evaluate the neurotoxicity induced by oligomeric tau proteins. These findings are in line with the hypothesis that tau oligomers are more toxic than tau aggregates to neuronal cells [32, 33]. By contrast, treatment of zebrafish embryos with gene products or chemical compounds that exhibit anti-apoptotic, anti-oxidative, or neurotrophic effects could prevent the tau-GFP-expressing neurons from death. The accessibility of live imaging and chemical treatment in our zebrafish model will allow screening more drugs against tauopathy.

## Methods

### Zebrafish care

Zebrafish embryos were raised at 28.5 °C, and different developmental stages were determined based on the criteria described in the Zebrafish Book [34]. All animal procedures were approved by the Academia Sinica Institutional Animal Care and Utilization Committee (ASIACUC) (protocol #10-12-114).

### Cloning of full-length cDNAs encoding z3R-tau and h4R-tau

Full-length cDNAs encoding z3R-tau and h4R-tau were isolated by PCR amplification using gene-specific primers (zTau-F, 5′-ATG GAC CAT CAG GAC CAC ATG AAT TCT-3′ and zTau-R, 5′-CAG GCC TTG TTT AGC AAG GGA GGC CGA-3′; hTau-F, 5′-ATG GCT GAG CCC CGC CAG GAG-3′ and hTau-R, 5′-CAA ACC CTG CTT GGC CAG GGA-3′) based on the sequences of zebrafish EST clones (accession numbers EH433182 and EH608572) or GenBank accession number BC114504. The cDNA encoding z3R-tau or h4R-tau was subcloned into pHA-YUN-GFP vector to generate pCMV-z3R-tau-GFP or pCMV-h4R-tau-GFP, respectively.

### Construction of expression plasmids

Complementary DNA encoding z3R-tau-Δ260, z3R-tau-Δ290, h4R-tau-Δ422, or h4R-tau-Δ392 was re-amplified by PCR using primers with built-in restriction sites; the resulting PCR products were then individually subcloned into pHA-YUN-GFP at appropriate sites to generate pCMV-z3R-tau-Δ260-GFP, pCMV-z3R-tau-Δ290-GFP, pCMV-h4R-tau-Δ422-GFP, and pCMV-h4R-tau-Δ392-GFP, respectively. To express GFP fusion proteins in neurons, each DNA fragment encoding a GFP-fusion protein was inserted into the corresponding sites of pHuC-GFP plasmid to replace the GFP coding region, thereby generating pHuC-z3R-tau-GFP, pHuC-z3R-tau-Δ260-GFP, pHuC-z3R-tau-Δ290-GFP, pHuC-h4R-tau-GFP, pHuC-h4R-tau-Δ422-GFP, and pHuC-h4R-tau-Δ392-GFP, respectively. The control plasmid pHuC-GFP was previously described, and GFP genes were driven by a zebrafish neuron-specific HuC promoter [31].

Expression plasmids pHuC-zBcl2-L1-HA-2A-mCherry, pHuC-zNrf2-HA-2A-mCherry, pGFAP-zBDNF-HA, and pGFAP-zGDNF-HA were constructed for co-injection with pHuC-z3R-tau-GFP or pHuC-h4R-tau-GFP. The zBcl2-L1, zNrf2, zBDNF, and zGDNF constructs were amplified based on sequences from NCBI GenBank accession numbers NM_131807 [35], NM_182889 [36], NM_001308649 [37] and NM_131732 [38], respectively, and then subcloned into pCMV-HA-2A-mCherry or pCMV-HA. The zBcl2-HA-2A-mCherry and zNrf2-HA-2A-mCherry sequences were inserted into the corresponding sites of the pHuC-GFP plasmid to replace the GFP coding region and to generate pHuC-zBcl2-L1-HA-2A-mCherry and pHuC-zNrf2-HA-2A-mCherry, respectively. The zBcl2-L1 or zNrf2 and mCherry proteins can be expressed from a single transcript by using a self-cleaving 2A peptide derived from porcine teschovirus-1 (P2A; GSGATNFSLLKQAGDVEENPGP) [39]. The zBDNF-HA and zGDNF-HA sequences were individually inserted into the pGFAP-GFP plasmid to replace the GFP coding region and to generate pGFAP-zBDNF-HA and pGFAP-zGDNF-HA, respectively. The control plasmid pGFAP-GFP was previously described, and GFP genes were driven by a zebrafish glial cell-specific GFAP promoter [40].

### Microinjection of zebrafish embryos

Plasmid DNA was injected into one-cell zygotes using a microinjection system consisting of a SZX9 stereomicroscope (Olympus, Tokyo, Japan) and an IM300 Microinjector (Narishige, Tokyo, Japan). The concentration of all plasmid DNA used to microinject is about 500 ng/μl and the amount of all plasmid DNA injected into zebrafish embryos is about 0.2 ng. Embryos at 24 and 48 h post-fertilization (hpf) were observed under an Olympus IX70-FLA inverted fluorescence microscope. Images were taken using the SPOT system (Diagnostic Instruments, Sterling Heights, MI).

### TUNEL assay

For detection of apoptotic cells, the embryos were fixed in 4 % PFA overnight, washed several times with PBST, and stored in methanol at −20 °C. To perform TUNEL assay, embryos were rehydrated with methanol/PBST series, treated with proteinase K, and then fixed in 4 % PFA at room temperature. Apoptotic cells were detected by In Situ Cell Death Detection Kit (Roche Diagnostics, Germany) according to instructions of the manufacturer.

### Whole-mount immunostaining

Whole-mount immunostaining was performed following standard protocols as previously described [41] with some modifications. The antibodies used were as follows: mouse anti-GFP (1E4) (1:300) (MBL, Nagoya, Japan), mouse anti-HA (F-7) (1:100) (Santa Cruz Biotechnology, Inc., Santa Cruz, CA), mouse anti-human PHF-Tau (AT8) (1:100) (Thermo Fisher Scientific, West Palm Beach, FL), rabbit anti- Caspase-9 (Novus Biologicals, Inc., Littleton, CO,USA), rabbit anti-Tau [pT212] (Thermo Fisher Scientific), rabbit anti-GFP (1:300) (Abcam, Cambridge, UK), Cy3-conjugated anti-mouse IgG (1:100), Cy2-conjugated anti-mouse IgG (1:100), Cy2-conjugated anti-rabbit IgG (1:100) and Cy3-conjugated anti-rabbit IgG (1:100) (Jackson ImmunoResearch Laboratories, Inc., West Grove, PA). High resolution images of the samples were captured using a Leica SP5 X Inverted Confocal Microscope.

### Western blot analysis

One hundred pHuC-h4R-tau-GFP-injected embryos or wild-type embryos were collected at 24 hpf for independent experiments. The sample preparation was based on the protocols described in the Zebrafish Book [34]. Clarified lysates containing equivalent amounts of proteins derived from zebrafish embryos were analyzed by Western blotting with the following antibodies: mouse anti-human PHF-Tau (AT8) (1:3000) (Thermo Fisher Scientific), rabbit anti-GFP (1:5000) (Abcam), mouse anti-GAPDH (1D4) (1:5000) (Novus Biologicals, Inc., Littleton, CO,USA), HRP-conjugated AffiniPure goat anti-mouse IgG (1:20000) (Jackson ImmunoResearch Laboratories, Inc., West Grove, PA).

### Chemical treatment

Stock solutions of DADS (10 mM) (Sigma) [42] and Luteolin (100 mM) (Sigma) [43] were prepared in DMSO. Zebrafish embryos injected with pHuC-h4R-tau-GFP were incubated in water containing DMSO or the above compounds from 6 to 48 hpf. The working concentration of DADS is 20 μM and the luteolin is 400 μM.

### Neuronal toxicity assay

The ability of wild-type human Tau protein to induce cell death in developing neuronal cells was investigated using pHuC-h4R-tau-GFP. The expression construct was injected into zebrafish embryos at the 1-cell stage. GFP-labeled neuronal cells were observed at 24 and 48 hpf under a fluorescence microscope. Certain GFP signals were observed in neuronal cells of 24 hpf embryos, before diminishing into small dots in 48 hpf embryos. However, other GFP signals remained intact in neuronal cells. The numbers of neurons with GFP signals in 48 hpf embryos were counted and separated into two groups: 2 or fewer neurons (0–2) and more than 2 neurons (between 3–5).

## Results

### Induction of neuronal death by overexpression of wild-type and truncated forms of human and zebrafish tau proteins in zebrafish embryo

Truncation of Tau protein by caspases and other proteases has been identified at discrete sites, including Asp^421^ [15–17, 44] and Glu^391^ [10, 45]. Purified recombinant Tau proteins truncated after Glu^391^ or Asp^421^, designated as h4R-tau-∆392 or h4R-tau-∆422, are prone to aggregate in vitro, resulting in faster rates of fibrillization than those of WT proteins [17, 18]. In addition, the caspase-3-cleaved tau fragment was demonstrated to exert toxic effects in cultured neural cells [16, 17, 46].

**Fig. 1:**
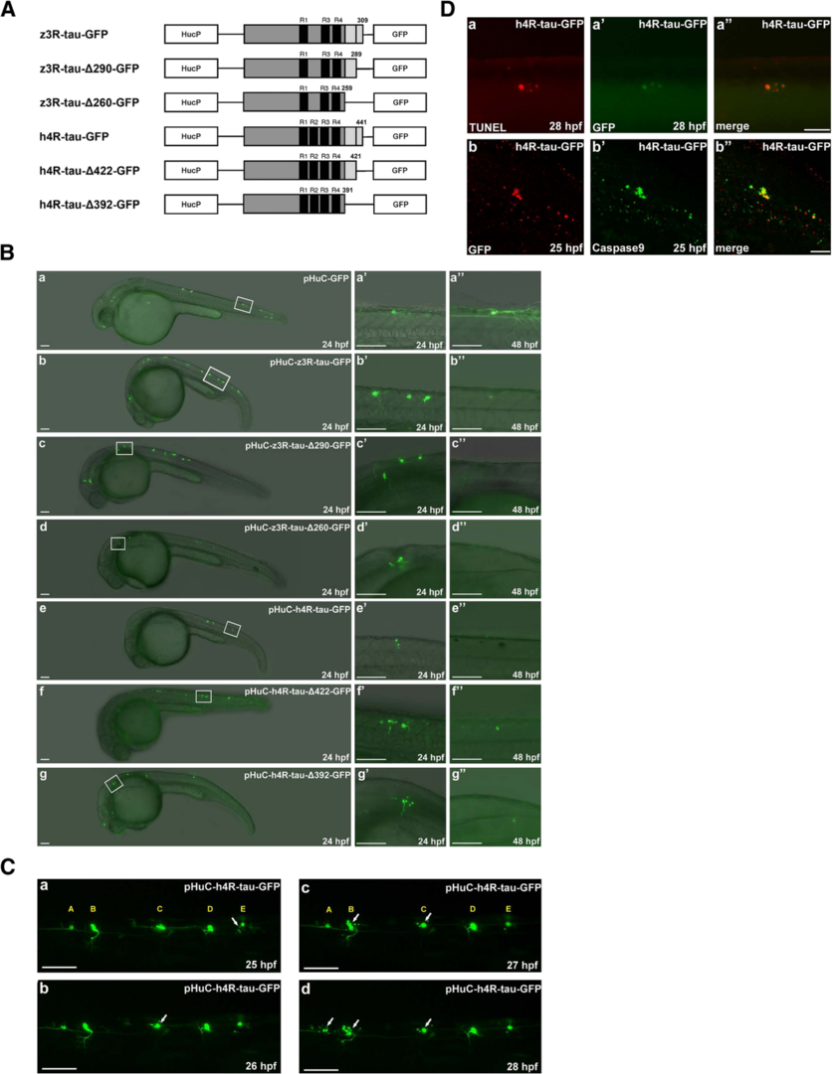
Overexpression of human and zebrafish tau proteins in zebrafish embryo resulted in neuronal death. **a** Schematic diagrams of each expression construct containing either wild-type or truncated forms of human and zebrafish Tau tagged with green fluorescence protein (GFP). Each expression construct was driven by the HuC promoter. The black bar represents one repeat of tubulin binding domain. The wild-type human and zebrafish tau proteins contain four and three repeats, respectively. **b** Each expression construct was microinjected into zebrafish embryos at the one-cell stage. Zebrafish embryos with GFP signals at 24 to 48 hpf were selected for image analysis. Embryos are shown in the lateral view with the anterior to the left and dorsal to the top. The boxed region of each panel (*a*-*g*) is enlarged (*a’*-*g”*) to show the GFP-labeled neuronal cells in 24 to 48 hpf embryos from the lateral view. Scale bars: 100 μm. **c** The five GFP-labeled neuronal cells in embryos injected with pHuC-h4R-tau-GFP were traced with the aid of time-lapse recording. Puncta formation was observed in neuron E at 25 hpf, neuron C at 26 hpf, neuron B at 27 hpf, and neuron A at 28 hpf. Scale bars: 100 μm. **d** TUNEL staining (panel a) and double immunostaining of zebrafish embryos expressing h4R-tau-GFP at different developmental stages was performed using polyclonal antibody against Caspase 9 and monoclonal antibody against GFP (panel *b*). Scale bar: 50 μm

These findings prompted us to examine whether zebrafish Tau proteins truncated after Asp^259^ or Asp^289^ (equivalent to human tau truncation at Glu^391^ or Asp^421^) can induce neurotoxic effects. Truncated zebrafish 3R-tau proteins were generated and tagged with green fluorescence protein (GFP), and designated as z3R-Tau-∆260-GFP or z3R-tau-∆290-GFP (Fig. 1a). The h4R-tau-∆392-GFP and h4R-tau-∆422-GFP constructs were generated by a similar approach. GFP was also fused to wild-type zebrafish and human tau proteins to generate z3R-tau-GFP and h4R-tau-GFP, respectively (Fig. 1a).

To investigate whether the truncated and wild-type tau proteins could affect the survival of neuronal cells, the neuronal expression of recombinant tau was driven by a neuron-specific HuC promoter [31]. As shown in Fig. 1b, GFP-labeled neuronal cells and axons were observed at 24 and 48 hpf (panels *a*, *a’*, and *a”*) in embryos injected with pHuC-GFP as a control. On the other hand, while GFP-labeled neuronal cells were viable at 24 hpf in embryos injected with z3R-tau-GFP (Fig. 1b, panels *b*, *b’*, and *b”*), z3R-tau-∆290-GFP (Fig. 1b, panels *c*, *c’*, and *c”*), z3R-tau-∆260-GFP (Fig. 1b, panels *d*, *d’*, and *d”*), h4R-tau-GFP (Fig. 1b, panels *e*, *e’*, and *e”*), h4R-tau-∆422-GFP (Fig. 1b, panels *f*, *f’*, and *f”*), or h4R-tau-∆392-GFP (Fig. 1b, panels *g*, *g’*, and *g”*), such neurons were dramatically lost at 48 hpf (Fig. 1b, panels *b”*-*g”*). Some GFP signals readily dissipated into small puncta (panels *d’*, *e’*, *f’*, and *g’*). With the aid of time-lapse recording, five GFP-labeled neuronal cells in embryos injected with h4R-tau-GFP were traced (Fig. 1c). Puncta formation was first observed in neuron E at 25 hpf, followed by neuron C at 26 hpf, neuron B at 27 hpf, and neuron A at 28 hpf. Interestingly, only neuron D remained intact during the recording time, suggesting that only this neuron may have survived. In order to distinguish whether the puncta formation is due to cell death, not only due to reduced GFP expression, we performed TUNEL staining and immunostaining with anti-caspase 9 as shown in Fig. 1d. These data indiated that expression of tau-GFP induced neuronal death through apoptosis.

### Expression of zebrafish Bcl2-L1 can prevent induction of neuronal death by overexpression of human 4R-tau and zebrafish 3R-tau

We proceeded to investigate whether certain signaling factors can prevent neuronal death induced by overexpression of wild-type human and zebrafish tau proteins. We first tested the effect of anti-apoptotic factor, Bcl2-L1, which has been shown to regulate a caspase-3-dependent apoptotic mechanism during thyroid development in zebrafish [35]. The C-terminal end of Bcl2-L1 was tagged with HA peptide for detection of protein production. Both Bcl2-L1-HA and another mCherry red fluorescence protein, a variant of the Discosoma red (DsRed) protein [47], were co-expressed from a single transcript by the use of a self-cleaving 2A peptide [39]. Thus, the expression level of Bcl2-L1-HA and mCherry protein is equal.

**Fig. 2:**
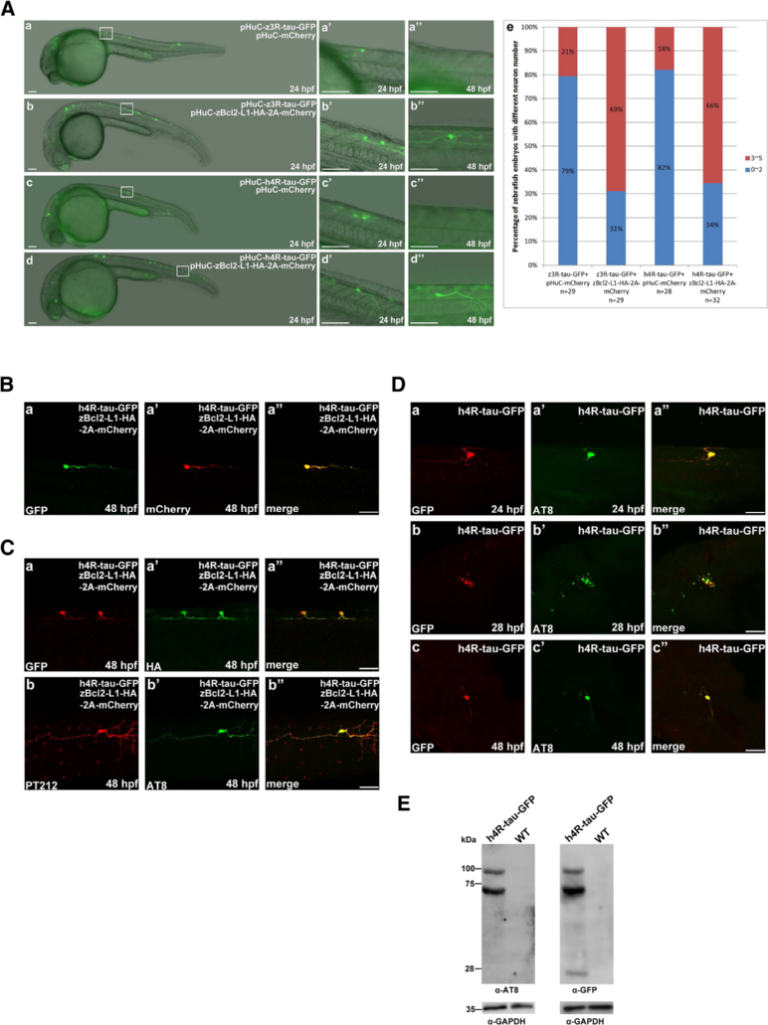
Zebrafish Bcl2-L1 overexpression prevented human 4R-tau-GFP and zebrafish 3R-tau-GFP induced Neuronal death. **a** GFP-labeled neuronal cells and axons were observed at 24 and 48 hpf in embryos co-injected with pHuC-zBcl2-L1-HA-2A-mCherry and pHuC-z3R-tau-GFP (*b*) or pHuC-h4R-tau-GFP (*d*). For comparison, embryos co-injected with pHuC-mCherry (panels *a* and *c*) were used as the control. The boxed regions are enlarged (*a’*-*d”*) to show the GFP-labeled neuronal cells in 24 and 48 hpf embryos from the lateral view. Scale bars: 100 μm. The protection effect of zBcl2-L1 against neuronal death by human tau-GFP or zebrafish tau-GFP was presented in panel e to show higher percentage, 69 % and 66 % of zebrafish embryos expressing zBcl2-L1 with more neuronal cells, compared to 21 % and 18 % without zBcl2-L1. **b** GFP signals (panel *a*) and mCherry signals (panel *a’*) in neuronal cells and axons in embryos co-injected with pHuC-h4R-tau-GFP and pHuC-zBcl2-L1-HA-2A-mCherry were colocalized (panel *a”*). Scale bar: 50 μm. **c** Double immunostaining of h4R-tau-GFP (GFP antibody, panel a) and Bcl2-L1-HA (HA antibody, panel *a’*) in spinal cord neurons of the aforementioned zebrafish embryos. The phosphorylation state of h4R-tau-GFP was detected using antibody pT212 (panel *b*) and antibody AT8 (panel *b’*). Embryos are shown from the lateral view with the anterior to the left and dorsal to the top. Scale bar: 50 μm. **d** Double immunostaining of zebrafish embryos expressing h4R-tau-GFP at different developmental stages was performed using polyclonal antibody against GFP and monoclonal antibody AT8. Scale bar: 50 μm. **e** Western blot analysis of total protein extract of zebrafish embryos expressing h4R-tau-GFP at 24 hpf was performed using polyclonal antibody against GFP and monoclonal antibody AT8

We demonstrated that GFP-labeled neuronal cells and axons could be observed at 24 and 48 hpf in embryos co-injected with either pHuC-zBcl2-L1-HA-2A-mCherry plus pHuC-z3R-tau-GFP (Fig. 2a, panels *a*, *a’*, *a”*) or pHuC-zBcl2-L1-HA-2A-mCherry plus pHuC-h4R-tau-GFP (Fig. 2a, panels *b*, *b’*, and *b”*). The protection effect of zBcl2-L1 was presented in panel e to show higher percentage, 69 % or 66 % of zebrafish embryos expressing zBcl2-L1 with more neuronal cells, compared to 21 % or 18 % without zBcl2-L1. These data suggested that overexpression of Bcl2-L1 in neurons can prevent neurotoxicity induced by zebrafish or human tau-GFP. The viable GFP-labeled neuron at 48 hpf not only displayed mCherry red fluorescence (Fig. 2b, panels *a* and *a’*), but was also immunoreactive to antibodies pT212 (Fig. 2c, panel *b*) and AT8 (Fig. 2c, panel *b’*). Antibody AT8 recognizes phosphorylated paired helical filament of human tau protein at both serine 202 and threonine 205. Antibody pT212 was used to detect specific phosphorylation of tau at T212, which has been proposed to play important roles in self-assembly of human tau protein.

To investigate the phosphorylation status of neuronal cells expressing only h4R-tau-GFP, we collected injected embryos at different time to perform double immunostaining. As shown in Fig. 2d, the injected embryo at 24 hpf expressed tau-GFP with positive reaction to antibody AT8 (panel *a’*). In 28 hpf embryo, the puncta was observed with positive signal to both GFP and AT8 (panels *b*, *b’* and *b”*). The remaining neuronal cell at 48 hpf expressed tau-GFP with positive signal to AT8 (panel *c’*). We also collected injected embryos at 24 hpf to perform Western blot analysis as shown in Fig. 2e. Monomeric and dimeric forms of tau-GFP were detected with positive reaction to antibody AT8.

Taken together, our data demonstrate that overexpression of Bcl2-L1 in neurons can effectively suppress the neurotoxicity induced by tau-GFP aggregates without disrupting the formation of phosphorylated paired helical filaments.

### Expression of zebrafish Nrf2 can prevent neuronal death induced by overexpression of human 4R-tau and zebrafish 3R-tau

**Fig. 3:**
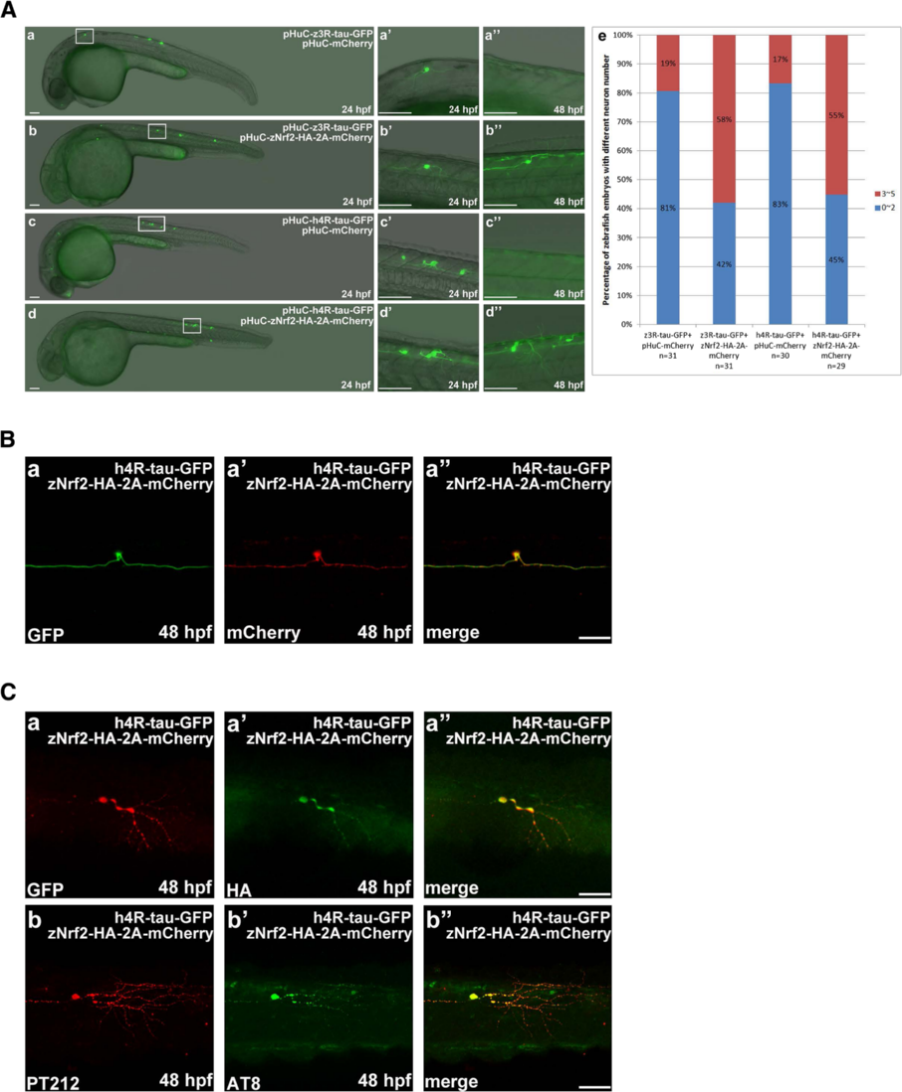
Zebrafish Nrf2 overexpression prevented human 4R-tau-GFP and zebrafish 3R-tau-GFP induced Neuronal death. **a** GFP-labeled neuronal cells and axons were observed at 24 and 48 hpf in embryos co-injected with pHuC-zNrf2-HA-2A-mCherry and pHuC-z3R-tau-GFP (*b*) or pHuC-h4R-tau-GFP (*d*). For comparison, embryos co-injected with pHuC-mCherry (panels *a* and *c*) were used as the control. The boxed regions are enlarged (*a’*-*d”*) to show the GFP-labeled neuronal cells in 24 and 48 hpf embryos from the lateral view. Scale bars: 100 μm. The protection effect of zNrf2 against neuronal death induced by human tau-GFP or zebrafish tau-GFP was presented in panel e to show higher percentage, 58 % and 55 % of zebrafish embryos expressing zNrf2 with more neuronal cells, compared to 19 % and 17 % without zNrf2. **b** GFP signals (panel *a*) and mCherry signals (panel *a’*) in neuronal cells and axons in embryos co-injected with pHuC-h4R-tau-GFP and pHuC-zNrf2-HA-2A-mCherry were colocalized (panel *a”*). Scale bar: 50 μm. **c** Double immunostaining of h4R-tau-GFP (GFP antibody) and zNrf2-HA (HA antibody, panel *a’*) in spinal cord neurons of the aforementioned zebrafish embryos. The phosphorylation state of h4R-tau-GFP was detected using antibody pT212 (panel *b*) and antibody AT8 (panel *b’*). Scale bar: 50 μm

Nuclear factor erythroid 2-related factor 2 (Nrf2) has been shown to be an important transcription factor in the defense against oxidative stress. Nucleus-localized Nrf2 binds to a conserved DNA motif called antioxidant response elements (ARE) to initiate the transcription of cytoprotective genes (phase II genes) [48]. Thus, the Nrf2-ARE pathway controls the majority of antioxidant pathways, including the synthesis of glutathione (GSH) and the expression of heme oxygenase-1 (HO-1). Activation of the Nrf2 − ARE pathway has been proposed to be a promising therapeutic approach for the treatment of neurodegenerative disorders [49, 50]. In addition, Nrf2 was recently identified as a possible target for AD treatment, due to its anti-oxidative abilities against Aβ-mediated neurotoxicity in vitro [51]. These results prompted us to investigate whether activation of anti-oxidative signaling can also suppress neurotoxicity elicited by overexpression of tau-GFP. Here, the C-terminal end of Nrf2 was tagged with HA peptide for detection, and Nrf2-HA was co-transcribed with mCherry (the two coding regions were linked with a sequence encoding a self-cleaving 2A peptide). Zebrafish embryos were co-injected with pHuC-zNrf2-HA-2A-mCherry and either pHuC-z3R-tau-GFP (Fig. 3a, panels *a*, *a’*, *a”*) or pHuC-h4R-tau-GFP (Fig. 3a, panels *b*, *b’*, and *b”*) at the 1-cell stage; GFP-labeled neuronal cells and axons were then imaged at 24 and 48 hpf. The viable GFP-labeled neuron at 48 hpf not only displayed mCherry-emitted red fluorescence (Fig. 3b, panels *a* and *a’*), but was also immunoreactive to antibodies pT212 (Fig. 3c, panel *b*) and AT8 (Fig. 3c, panel *b’*). These data clearly indicate that overexpression of Nrf2 can protect against tau-GFP-elicited neurotoxicity downstream of tau-GFP hyperphosphorylation and aggregation.

### Neuronal death by overexpression of human 4R-tau and zebrafish 3R-tau can be rescued by expression of zebrafish GDNF

Neurotrophins (NTs) are important for the survival and maintenance of specific neuronal populations in the brain. These NTs in mammals include nerve growth factor (NGF), brain-derived neurotrophic factor (BDNF), neurotrophin-3 (NT-3), and NT-4/5 [52, 53]. BDNF deficiency has been implicated in the pathogenesis of Huntington's disease (HD) [54, 55]. Glial cell-derived neurotrophic factor (GDNF) is another potent NT for a variety of neuronal populations [56]. Recently, it has been shown to have therapeutic potential for neurodegenerative disorders, including AD [57] and Parkinson disease (PD) [58].

**Fig. 4:**
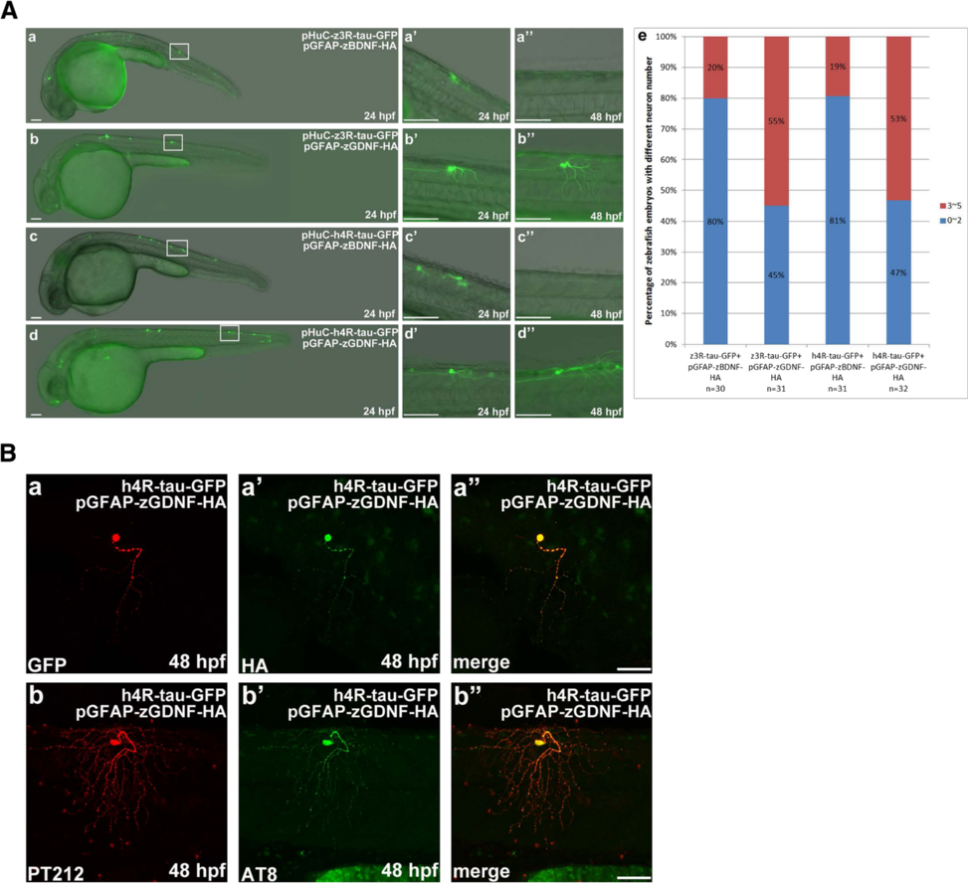
Zebrafish GDNF overexpression prevented human 4R-tau-GFP and zebrafish 3R-tau-GFP induced Neuronal death. **a** GFP-labeled neuronal cells and axons were observed at 24 and 48 hpf in embryos co-injected with pGFAP-zGDNF-HA and pHuC-z3R-tau-GFP (*b*) or pHuC-h4R-tau-GFP (*d*). For comparison, embryos co-injected with pGFAP-zBDNF-HA (panels *a* and *c*) were used as the control. The boxed regions are enlarged (*a’*-*d”*) to show the GFP-labeled neuronal cells in 24 to 48 hpf embryos from the lateral view. Scale bars: 100 μm. The protection effect of GDNF against neuronal death induced by human tau-GFP or zebrafish tau-GFP was presented in panel *e* to show higher percentage, 55 % and 53 % of zebrafish embryos expressing GDNF with more neuronal cells, compared to 20 % and 19 % without GDNF. **b** Double immunostaining of h4R-tau-GFP (GFP antibody, panel *a*) and GDNF-HA (HA antibody, panel *a’*) in spinal cord neurons of the aforementioned zebrafish embryos. The phosphorylation state of h4R-tau-GFP was detected using antibody pT212 (panel *b*) and antibody AT8 (panel *b’*). Scale bar: 50 μm

Here, we further investigated whether certain NTs can prevent neuronal death induced by overexpression of wild-type human and zebrafish tau proteins. The C-terminal ends of BDNF [59] and GDNF [60] were tagged with HA peptide for easy detection of protein production. We expressed BDNF-HA or GDNF-HA under the control of a glia-specific glial fibrillary acidic protein (GFAP) gene promoter [40] to examine whether these proteins are able to promote the survival of neuronal cells expressing human and zebrafish tau-GFP proteins. Consistent with the reported expression profile of GFAP in a transgenic zebrafish model [40], zebrafish embryos that were co-injected with pGFAP-zGDNF-HA and either pHuC-z3R-tau-GFP (Fig. 4a, panels *b*, *b’*, *b”*) or pHuC-h4R-tau-GFP (Fig. 4a, panels *d*, *d’*, and *d”*) contained GFP-labeled neuronal cells and axons at 24 and 48 hpf. The protection effect of GDNF against neuronal death induced by human tau-GFP or zebrafish tau-GFP was presented in panel e to show higher percentage, 55 % or 53 % of zebrafish embryos expressing GDNF with more neuronal cells, compared to 20 % or 19 % without GDNF. The viable GFP-labeled neuron at 48 hpf not only displayed immunoreactivity to HA (Fig. 4b, panels *a* and *a’*), but also showed positive immunoreactivity to antibodies pT212 (Fig. 4b, panel *b*) and AT8 (Fig. 4b, panel *b’*). These data indicate that zGDNF-HA can possibly bind to GDNF family receptor subtype α1 (GFRα1) to induce neuroprotective effects in tau-GFP-expressing neurons, without interfering with the hyperphosphorylation and aggregation of tau-GFP protein. In contrast, BDNF-HA expression in zebrafish embryos (Fig. 4a, panels *a* and *c*) was not sufficient to suppress tauopathy-elicited neurotoxicity.

### Treatment with DADS and luteolin can prevent neuronal death induced by overexpression of human 4R-tau

**Fig. 5:**
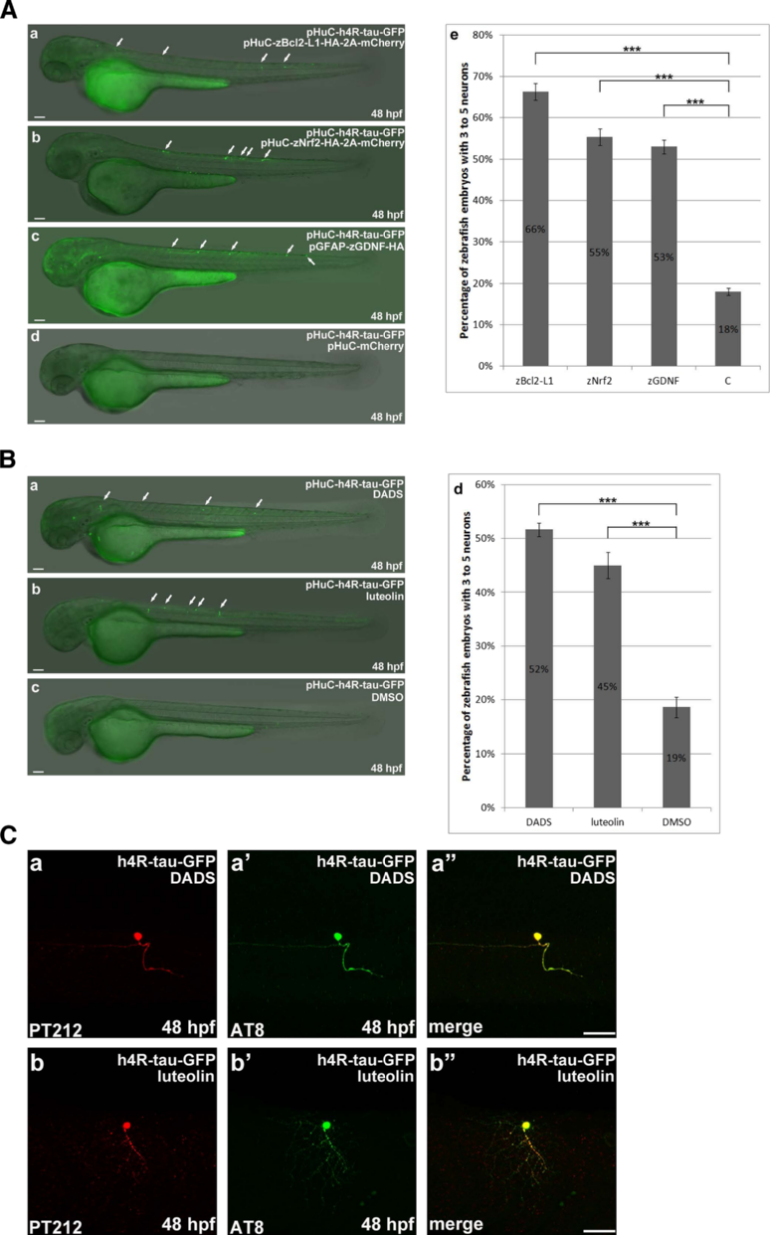
DADS and luteolin treatment prevent neuronal death induced by overexpression of h4R-tau-GFP. **a** In pHuC-h4R-Tau-GFP-injected embryos, which respectively co-expressed Bcl2-L1 (panel *a*), Nrf2 (panel b), or GDNF (panel *c*), there are more neuronal cells survived. Statistical analysis (panel *e*) represents the quantitative results of zebrafish embryos respectively co-expressing Bcl2-L1 or Nrf2 or GDNF to have higher percentage of more neuronal cells compared to the control. The n value is indicated. **b** Numbers of zebrafish embryos with more neuronal cells were counted as described above for pHuC-h4R-Tau-GFP-injected embryos treated with DADS (diallyl-disulfide) (panel *a*) and luteolin (panel *b*). Statistical analysis (panel *e*) was presented similarly as described above to show that pHuC-h4R-Tau-GFP-injected embryos treated with DADS or luteolin have higher percentage of more neuronal cells compared to the control. **c** The effects of DADS and luteolin treatment on h4R-tau-GFP-induced neuronal death were confirmed by double immunostaining of GFP-labeled neurons at 48 hpf. The phosphorylation state of h4R-tau-GFP was detected by antibody pT212 (panels *a* and *b*) and antibody AT8 (panels *a’* and *b’*). Embryos are shown from the lateral view with the anterior to the left and dorsal to the top. Scale bar: 50 μm

To further confirm the hypothesis that augmentation of anti-oxidative pathways can suppress tauopathy-induced neurotoxicity, we examined neuronal survival in zebrafish embryos co-injected with pHuC-h4R-Tau-GFP and pHuC-zBcl2-L1-HA-2A-mCherry, pHuC-zNrf2-HA-2A-mCherry, pGFAP-zGDNF-HA, or pHuC-mCherry at 48 hpf. Viable GFP-positive neurons in individual embryos were counted. Injected zebrafish embryos at 48 hpf were categorized into two groups: those with 2 or fewer viable GFP-positive neurons per embryo (0 ~ 2) and those with more than 2 GFP-positive neurons per embryo (3 ~ 5). We found that 82 % of embryos injected with pHuC-h4R-Tau-GFP had only 0–2 GFP-positive neurons, while the other 18 % contained more than 2 GFP-positive neurons (Fig. 5a, panel *e*). We observed that co-expression of Bcl2-L1, Nrf2, or GDNF significantly promoted the survival of GFP-positive neurons in embryos injected with pHuC-h4R-Tau-GFP, increasing the percentage of embryos containing more than 2 GFP-positive neurons to 66 %, 55 %, and 53 %, respectively (Fig. 5a, panel *e*). Consistent with these findings, tau-GFP-expressing embryos that were treated with diallyl-disulfide (DADS) or luteolin also exhibited significant enhancement of neuronal survival, increasing the percentage of embryos containing more than 2 GFP-positive neurons to 52 % and 45 %, respectively (Fig. 5b, panel *d*). DADS has been reported to activate the Nrf-2/HO-1 pathway [42], while luteolin has been identified to be highly active in inducing the synthesis and secretion of neurotrophic factors, including GDNF [43]. However, luteolin also has been identified to provide neuroprotective effects, possibly through activation of the Nrf2–ARE pathway [61]. These data suggest that chemical stimulation of anti-oxidative signaling by DADS or luteolin can recapitulate the neuroprotective effects induced by the overexpression of Nrf2 (Fig. 5b, panel *d* versus Fig. 5a, panel *e*). Immunostaining of tau-expressing zebrafish embryos with AT8 and pT212 antibodies further confirmed that DADS-induced and luteolin-induced stimulation of anti-oxidative signaling can suppress neurotoxicity despite the presence of hyperphosphorylated and aggregated tau (Fig. 5c, panel *a*-*b”*). Together, our present findings strongly suggest that the newly established zebrafish models of tauopathy are highly conducive to high-content live imaging analysis, thereby facilitating the discovery of novel anti-tauopathy drugs and therapeutics against AD.

## Discussion

In this study, a transient expression system was established to express GFP fusion proteins of either zebrafish 3R-tau or human 4R-tau under the control of a neuron-specific HuC promoter [31]. In this system, approximately 10 neuronal cells expressing tau-GFP in zebrafish embryos were directly imaged and traced by time-lapse recording to evaluate the neurotoxicity induced by tau-GFP proteins. As shown in Fig. 1c, five GFP-labeled neuronal cells in embryos injected with pHuC-h4R-tau-GFP were traced from 25 hpf to 28 hpf, but only one neuron appeared to be intact. This observation is consistent with the finding that 81 % of embryos injected with pHuC-h4R-Tau-GFP had only 0–2 neurons (Fig. 5a, panel *e*). It is also consistent with an earlier proposal that tau oligomers, rather than tau aggregates, are more toxic to the cell [32, 33].

Truncation of tau protein by caspases and other proteases has been previously reported; purified recombinant human tau proteins truncated after Glu^391^ or Asp^421^ (equivalent to h4R-tau-△392 and h4R-tau-△422 in this study) are prone to aggregation in vitro, resulting in faster rates of fibrillization [17, 18]. Initially, we examined whether forms of zebrafish tau protein truncated after Asp^259^ or Asp^289^ (equivalent to human tau truncation at Glu^391^ or Asp^421^) elicit more neurotoxic effects than that of wild-type. Wild-type human tau-GFP exerts a strong neurotoxic effect (Fig. 1c; Fig. 5a, panel *e*); our data (Fig. 1b) indicates that truncated forms of either zebrafish or human tau protein induce similar neurotoxic effects to that of wild-type. Nrf2 has been shown to play pivotal roles in the defense against oxidative stress. In normal cells, the protein Kelch-like ECH-associated protein 1 (Keap1) forms a complex with Nrf2 through its active sulfhydryl group of cysteine residues. This association anchors Nrf2 in cytosol and directs it to ubiquitin-mediated proteasome degradation [62]. Electrophilic agents, such as gracilins [63] and DADS [42], can modify Keap1 to prevent it from targeting Nrf2 for degradation, thereby promoting Nrf2 stabilization and subsequent activation of Nrf2 target genes. Nrf2 was recently identified as a possible target for AD treatment, due to its antioxidative protective abilities against Aβ-mediated toxicity in vitro [51]. Dimethyl fumarate (DMF) is another synthetic Nrf2 activator that has been approved by the FDA for treatment of multiple sclerosis [64]. In this study, Nrf2 overexpression and DADS treatment were shown to effectively protect neuronal cells expressing tau-GFP from death. Thus, our expression system could be used to evaluate the therapeutic potential of other electrophilic agents, such as gracilins and DMF.

GDNF was first identified as a trophic factor for embryonic midbrain dopaminergic neurons [65], and subsequently found to act as a potent NT for a variety of neuronal populations [56], including peripheral neurons such as enteric, sympathetic, and parasympathetic neurons [66]. For intracellular signaling, GDNF first binds to glycosylphosphatidyl inositol (GPI)-anchored GDNF-family receptor α1 (GFRα1), and then recruits a transmembrane receptor RET [67] to form a complex that enables autophosphorylation of RET, which in turn initiates a number of downstream intracellular pathways [68]. However, GDNF can also signal independently of Ret through neural cell adhesion molecule (NCAM) or interacting with heparin sulphate glycosaminoglycans [69].

GDNF is increasingly recognized to be a potent neurotrophic factor with therapeutic potential against neurodegenerative diseases, including AD. Recombinant lentiviral vectors were previously used to overexpress the GDNF gene in hippocampal astrocytes of 3xTg-AD mice, revealing that GDNF exerts neuroprotective effects in this AD experimental model [70]. In the present study, zebrafish GDNF was expressed under a glia-specific GFAP gene promoter [40], which conferred neuroprotection against tau-GFP-induced neuronal death (Fig. 4a and b). In addition to the therapeutic potential for AD [57] and PD [58], this is the first report to indicate that GDNF has therapeutic potential for treating tau-induced neuronal death. In addition, chemical compounds have been shown to have the potential to stimulate synthesis and secretion of BDNF and GDNF in cultured astrocytes [71]. Although luteolin has been identified to be highly active at inducing the synthesis and secretion of neurotrophic factors, including GDNF [43], luteolin has also been identified to provide neuroprotective effects, possibly through activation of the Nrf2–ARE pathway [61].

## Conclusions

In conclusion, our zebrafish tauopathy models can be subjected to high-content live imaging analysis, making them an ideal platform with which to identify anti-tauopathy drugs and treatments against AD.

## Abbreviations

AD: alzheimer’s disease
ARE: antioxidant response elements
BDNF: brain-derived neurotrophic factor
DADS: diallyl-disulfide
DMF: dimethyl fumarate
DsRed: discosoma red
FTD: frontotemporal dementia
FTDP-17: frontotemporal dementia with parkinsonism linked to chromosome-17
GDNF: glial cell-derived neurotrophic factor
GFAP: glial fibrillary acidic protein
GFRα1: GDNF-family receptor α1
GSH: glutathione
HD: huntington’s disease
HO-1: heme oxygenase-1
Hpf: hours post-fertilization
Keap1: Kelch-like ECH-associated protein 1
MAPT: microtubule-associated protein tau
MT: microtubule
NCAM: neural cell adhesion molecule
NFTs: neurofibrillary tangles
NGF: nerve growth factor
Nrf2: nuclear factor erythroid 2-related factor 2
NT: neurotrophin
NT-3: neurotrophin-3
PD: parkinson disease
